# The Role of Strigolactones in Nutrient-Stress Responses in Plants

**DOI:** 10.3390/ijms14059286

**Published:** 2013-04-29

**Authors:** Marek Marzec, Aleksandra Muszynska, Damian Gruszka

**Affiliations:** 1 Department of Genetics, Faculty of Biology and Environmental Protection, University of Silesia, Katowice 40-032, Poland; E-Mail: damian.gruszka@us.edu.pl; 2 Department of Physiology and Cell Biology, Leibniz Institute of Plant Genetics and Crop Plant Research (IPK), Gatersleben 06466, Germany; E-Mail: muszynska@ipk-gatersleben.de

**Keywords:** biosynthesis, nitrogen (N), phosphorus (P), mycorrhizae, nodulation, signaling, stress response, strigolactones (SLs)

## Abstract

Strigolactones (SLs) are a new group of plant hormones, which have been intensively investigated during the last few years. The wide spectrum of SLs actions, including the regulation of shoot/root architecture, and the stimulation of the interactions between roots and fungi or bacteria, as well as the stimulation of germination of parasitic plants, indicates that this group of hormones may play an important role in the mechanisms that control soil exploration, and the root-mediated uptake of nutrients. Current studies have shown that SLs might be factors that have an influence on the plant response to a deficiency of macronutrients. Experimental data from the last four years have confirmed that the biosynthesis and exudation of SLs are increased under phosphorus and nitrogen deficiency. All these data suggest that SLs may regulate the complex response to nutrient stress, which include not only the modification of the plant developmental process, but also the cooperation with other organisms in order to minimize the effects of threats. In this paper the results of studies that indicate that SLs play an important role in the response to nutrient stress are reviewed and the consequences of the higher biosynthesis and exudation of SLs in response to phosphorus and nitrogen deficiency are discussed.

## 1. Introduction

Strigolactones (SLs) are a new group of plant hormones that have been intensively investigated during the last few years. In the first report describing SLs from 1966, their role as stimulators of the germination of parasitic plants (*Orobranche* and *Striga*) was postulated [1]. Subsequently, SLs were identified as signaling molecules that play an important function during the stimulation of symbiosis between plant roots and arbuscular mycorrhizal fungi (AMF), mainly as a hyphal branching factor [2]. In the both cases, SLs operated outside of the plant. They were present in the root exudate that penetrated the soil and interacted with external organisms. However, the presence of SLs in the exudation of non-mycorrhizal species, such as *Arabiopsis thaliana*, indicated some additional functions of these hormones in plants [3]. A few years ago, an analysis of more branched mutants revealed the role of SLs in the regulation of the above-ground architecture of plants [4,5]. According to current knowledge, SLs may act as the secondary messenger activated by auxin [6,7] or higher concentration of SLs causes disorders in the export of auxin from the axillary buds and increases the auxin concentration, which then suppresses the outgrowth of buds [8].

Recent studies have allowed the participation of SLs in root and root hair development [9–12], mesocotyl elongation [13] and nodulation [14] to be described. Most of the knowledge about SLs and their role in plants comes from studies on the different mutants that are affected in SL biosynthesis or signaling. In the past few years, the homologues of the genes involved in SLs biosynthesis and signaling have been identified in many other species, including algae, such as *Chlamydomonas reinhardtii* or mosses, such as *Physcomitrella patens* [15–17].

**Figure 1:**
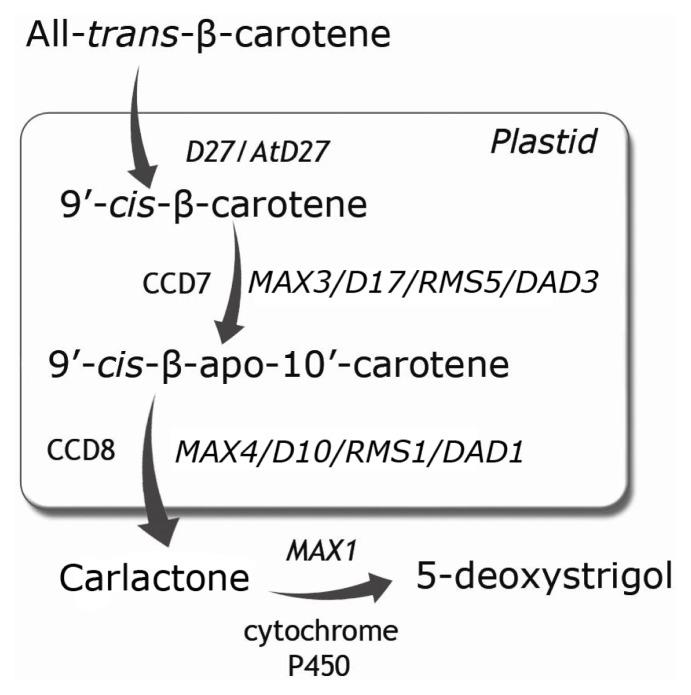
The biosynthesis pathway of Strigolactones (SLs). A detailed description is given in the text. According to [21,23].

SLs are secondary metabolites, which are synthetized from carotenoid precursors mainly in the roots [18]. In rice and Arabidopsis, all-*trans*-β-carotene is isomerized into 9′-*cis*-β-carotene by the iron-containing protein D27 in the plastids [19,20]. This step is followed by a reaction in which 9′-*cis*-β-carotene is oxidatively tailored by two carotenoid-cleavage dioxygenases (CCDs): CCD7 attacks the 9′ and 10′ bond of β-carotene and then CCD8 cleaves the obtained compounds into carlactone, which is the precursor of SLs [20]. Until 2012, the D27 protein was identified only in rice and its function was determined based on experiments carried out on the high-branched rice mutant *d27*, whose phenotype can be reversed by treatment with GR24, which is the synthetic analogue of SLs [19]. The Arabidopsis orthologue of D27 was characterized recently and detailed studies allowed the presented model of SLs biosynthesis to be proposed [20] (Figure 1). However, both CCDs have been identified in many different species [21] and the analysis of mutants carrying lesions in the genes that encode these enzymes have allowed the role of SLs in the regulation of shoot branching to be described [4,5]. The last stage in the biosynthesis of SLs is the conversion of carlactone into 5-deoxystrigol, one of the precursors of other SLs. This step of SLs biosynthesis is mediated by the enzymes from cytochrome P450 monooxygenase family and one already characterized representative of this protein family is encoded by the *MAX1*gene, identified in Arabidopsis [20,22] (Figure 1).

The perception and transduction pathways of SLs are still not well understood and only a few proteins have been implicated in these processes. The gene encoding proteins that are involved in the signaling pathway of SLs were characterized based on an analysis of highly branched mutants that were insensitive to GR24 treatment and contained a higher level of SLs in comparison to a wild-type [24]. The best characterized player of SLs perception is the leucine-rich F-box protein (MAX2/D3/RMS4), which has been described in many species (Table 1). It is suggested that this protein is a part of Skip-Cullin-F-box (SCF) ubiquitin ligase that mediates protein degradation [25,26]. SCF complexes play a crucial role in the signal transduction of other phytohormones, such as auxin, ethylene or jasmonic acid [27]. The second component of the RMS/MAX/D signaling pathway belongs to the α/β-hydrolase superfamily and may play role in the perception of SLs or the binding and conversion of SLs into a bioactive form [21]. The α/β-hydrolase is encoded by the *D14/D88/HTD2* genes identified e.g., in rice [28–31], Arabidopsis [31] and *Petunia hybrida* [26]. The crystal structure of the D14 protein was recently published, which allows a better understanding of the mechanisms of its action. It is now presumed that both proteins, D14 and F-box, directly interact during the perception of a SLs signal [32]. The binding of SL molecule by D3/MAX2 protein promotes its conformational alteration and allows the complex D3/MAX2-D14/DAD2 to be created [26,33]. It is suggested that the players that catalyze each stage of the RMS/MAX/D pathway are known [21]; however, new components such as a transporter of SLs are still being identified [34]. Protein PDR1, belonging to the ABC transporters, was characterized in petunia as involved in the long-distance transport of SLs from the root to the shoot and also in the root tissue. PDR1 plays an important role in axillary branching or AM symbioses, and *pdr1* mutant is defective in SLs exudation to the soil and presents more branched phenotype [34,35].

During the last four years, the role of SLs in the plant response to abiotic stresses has been investigated. Most of the research was carried on plants that were grown under phosphorous and nitrogen deficiency. In presented review the results of studies that indicate that SLs play an important role in the response to P and N starvation was described. Additionally the potential role of SLs in responses to other abiotic stresses was present.

## 2. SLs Biosynthesis and Exudation under Nutrient Stress Conditions

Phosphorus (P) is one of the most important macronutrients that is required for plant growth and development. It plays a crucial role in many metabolic processes and regulatory pathways, such as phosphorylation reactions, and it is also a structural component of fundamentally important macromolecules such as nucleic acids, ATP and membrane lipids [36]. Nitrogen (N), which can be found in different crucial macromolecules such as nucleic acids and amino acids, is another important macronutrient for plants. Although N makes up 78% of the volume of the atmosphere, plants cannot uptake it because two N atoms are strongly connected by a stable triple covalent bond [37]. This is why plants interact with symbiotic bacteria that can convert the atmospheric N into ammonium and then transfer it to the host plant [38]. The availability of both macronutrients, P and N, is crucial for plant development, and therefore a quick response to N/P-deficiency is required in order for a plant to survive.

Studies on different plant species have revealed that the level of macronutrients in the soil is a powerful regulator of the biosynthesis and exudation of SLs. The first reports about the correlation between nutrient deficiency and a higher secretion of SLs were published in 2007 [39,40]. The effect of deficiency of different nutrients on the biosynthesis and exudation of SLs was analyzed in *Trifolium pretense* L., which is known to be a host for the parasitic plants and AMF. In this case, the level of orobanchol was analyzed under low P, N, K, magnesium (Mg) and calcium (Ca) conditions. A higher production of orobanchol (20 times higher) was observed only under P deficiency, whereas the other analyzed nutrients did not affect the biosynthesis of SLs [39] (Table 2). Analyses of the level of SLs in the roots of *Sorghum bicolor* L. under P and N deficiency revealed that the production of 5-deoxystrigol, one of the major SLs identified in this species, was almost 30 times higher during the response to nutrient stress conditions than in the control plants [40] (Table 2). Additionally, almost a 30-fold increase of 5-deoxystrigol exudation was observed in sorghum roots in low N conditions. In the case of low P conditions, the exudation of 5-deoxystrigol was 20 times higher than in the control (Table 2). The higher secretion of SLs under nutrient stress conditions resulted in a more than 100 times stronger stimulation of the seed germination of the parasitic plant *Striga hermonthica* [40]. However, additional analyses revealed that a low level of other nutrients, such as potassium (K), did not affect the production and exudation of 5-deoxystrigol in sorghum. Moreover, the root exudate of plants grown under K deficiency did not stimulate the germination of parasitic seeds [40]. These results showed that low K conditions did not cause a higher biosynthesis and exudation of 5-deoxystrigol, which could be measured using liquid-chromatography coupled with tandem mass spectrometry (LC-MS/MS), but also that it did not affect the exudation of other SLs that might stimulate the germination of *S. hermonthica* seeds. Results obtained for sorghum and red clover indicated that the response of the exudation of SLs to nutrient availability varies across plant species. One of the hypotheses assumed that the observed differences were due to different strategies of macronutrient uptake in legumes (red clover) and non-legumes (sorghum) [39,40].

To confirm this hypothesis, Yoneyama and coworkers [41] tested the influence of P and N starvation on the production and exudation of SLs in six species from four different families: *Astragalus sinicus* L. (Chinese milk vetch) and *Medicago sativa* L. (alfalfa) from Fabaceae, *Lactuca sativa* L. (lettuce) and *Calendula officinalis* L. (marigold) from Asteraceae, *Solanum lycopersicum* L. from Solanaceae and *Triticum aestivum* L. from Poaceae. Two of these species, alfalfa and Chinese milk vetch, belong to legumes, whereas four others, lettuce, marigold, tomato and wheat, are non-legumes. As was expected, P deficiency induced the production and exudation of SLs in all of the analyzed species, whereas the effect of N starvation on the biosynthesis and exudation of SLs was not observed in alfalfa and, surprisingly, also not in tomato [41] (Table 2). These results showed that the production and exudation of SLs in response to a low level of N and/or P is not conserved across legumes and non-legumes. In the non-legume tomato, only P starvation induced a response based on the production of SLs, whereas in the legume plant, Chinese milk vetch, both analyzed stresses caused an increase in the biosynthesis of SLs [41] (Table 2). These results were recently confirmed by work published about another legume species, *Pisum sativum* L. [42]. The obtained results showed that during P starvation in pea, the level of fabacyl acetate and orobanchyl acetate, two of major SLs identified in this species, increased more than 10 times in comparison with control plants, whereas a low level of N caused a 3-fold increase of these compounds [42] (Table 2). All of the results from recent studies confirm that SLs are involved in the plant response to a macronutrient (P and/or N) deficiency, but they also show that this response is not solely dependent on whether the plant belongs to legumes or non-legumes [41,42] (Table 2).

An interesting aspect of the biosynthesis and exudation of SLs under macronutrient deficiency was discovered during experiments on rice [43,44]. As had been expected, exudates obtained from the roots of plants grown in low P and N conditions induced the germination of a higher number of *S. hermonthica* seeds than the root exudate of control plants [44] (Table 2). Moreover, LC-MS/MS analysis revealed a higher level of five SLs in the stressed plants. Three of these compounds have not yet been characterized, whereas two others, orobanchol and 2′-epi-5-deoxystrigol, are well-known SLs in rice [44]. These results indicate that the plant response to P and/or N starvation may include the higher production and exudation of many different SLs. An analysis of two rice cultivars (IAC 165 and TN 1) under P starvation showed differences in the case of the exudation of SLs [44] (Table 2). Under nutrient stress conditions, IAC 165 induced the germination of parasitic seeds of almost 50% and also produced a 100-fold higher amount of SLs than TN 1. However, no differences in the production of SLs were observed between cultivars when plants were grown in the control conditions [44]. These results indicate that there is a large variation in the production of SLs across cultivars and explains the differences in the results published for the same species. For example, under P deficiency a tomato plant cv. M82 increased the biosynthesis of orobanchol by 100-fold [41], whereas in a similar work on tomato plant cv. MoneyMaker increased production was reported not only for orobranchol, but also for solanacol and two or three unknown didehydro-orobanchol isomers [45] (Table 2). In addition, in the case of P deficiency, the highest production of SLs was observed in the experiment with the absence of this macronutrient (0%), whereas the strongest response to N deficiency was reported when only 25%–50% of the normal level of P was available to plants [44]. These results showed further differences in the uptake strategies in plants for different macronutrients.

The increased production of SLs in response to P and/or N deficiency has been described in many species, but the question of whether it is the result of a higher expression of the gene encoding proteins involved in the biosynthesis of SLs or the higher activity of proteins already present in a plant still remains. To answer this question, the expression of three rice genes involved in the production of SLs (*D10*, *D17* and *D27*) under nutrient stress conditions was measured [43]. An analysis of plants grown under low P for two weeks and then transferred to a medium containing the optimal concentration of this macronutrient revealed that the level of SLs in the roots of these plans decreased significantly during the first day after P supplementation and the expression of the analyzed genes correlated with P availability [43]. The expression of *D10*, *D17* and *D27* increased under P deficiency, whereas the expression of these genes decreased after P supplementation [43]. Moreover, in petunia, the expression of genes encoding PRD1, an ABC transporter protein that is involved in the long-distance transport of SLs, increased under P starvation [34]. All these data shed new light on the plant response to P starvation and explain the changes in the production and exudation of SLs under the nutrient stress conditions described. Higher SLs production and exudation is correlated with increased expression of genes encoding proteins involved in the biosynthesis and transport of SLs.

## 3. SLs Regulate the Above-Ground Architecture of Plants during a Response to Nutrient Stress Conditions

Shoot branching is correlated with many different external factors such as sunlight, water or plant density. However, the modification of shoot architecture has also been reported in the case of a P deficiency [46]. A low level of P in rice resulted in the inhibition of shoot growth and tiller production. The seedlings of plants grown for two weeks under P deficiency showed the inhibition of the outgrowth of tiller buds, which normally start to develop in control conditions [43]. In contrast, the seedlings of mutants with affected SL signaling (*d3*) and biosynthesis (*d10*) did not show this inhibition under a low level of P and the outgrowth of tiller buds was higher than in a wild-type grown in control conditions [43]. Similar results were obtained for Arabidopsis; in this case, a wild-type (Columbia-0) and three SL mutants, *max1*, *max2* and *max4*, were analyzed [35]. Under control conditions, the number of secondary rosette branches observed in the mutants was more than twice as high as that in Col-0, nine and four, respectively. However, when plants were grown in low P conditions, the differences in the number of secondary rosette branches between the mutants and the wild-type increased. In the case of Col-0, the number of secondary rosette branches was reduced, but in the case of all of the mutants they remained unchanged [35]. These results clearly indicate that SLs mutants are unable to regulate shoot branching in response to P starvation.

But why does the plant strategy to survive under a P deficiency include decreasing shoot branching? The answer could be that plants under a low P level have to limit the production of new tillers/branches in order to protect the required minimum amount of this macronutrient for the shoots that have already developed. The SLs produced during stress response play a key role in the inhibition of branching under P starvation. The lack of the correlation between the P concentration and the number of tillers/axillary branches in the mutants with affected SL biosynthesis and signaling clearly showed that both the production and perception of SLs are required for the suppression of tiller bud outgrowth under a P deficiency [35,47]. Additionally, as was mentioned, a higher expression of the gene encoding transporter of SLs was described for petunia plants that were grown in low P conditions [34]. Also in Arabidopsis and tomato was showed that under P deficiency the transport of SLs trough xylem was increased [35]. This allows to assumption that in a response to a low level of P, the transport of SLs from the roots to the shoot increases. This means that plant strategy to survive under a low P level is mediated by SLs and that this class of hormones may play a primary role in the response to nutrient stress conditions.

## 4. SLs Regulate Root Development under Nutrient Stress Conditions

As was already mentioned, P is immobile in the soil and for the uptake of higher amounts of this macronutrient, plant roots have to explore new areas in the soil. This is why the P concentration influences the root system architecture; in the case of a low level of this macronutrient a higher number of longer lateral roots (LRs) is present, while the development of primary roots (PRs) is repressed [48]. Moreover, the number of root hairs (RHs) and their length increases, which allows a plant to uptake more P due to an increase in the contact-surface between the root system and the soil [48,49].

**Figure 2:**
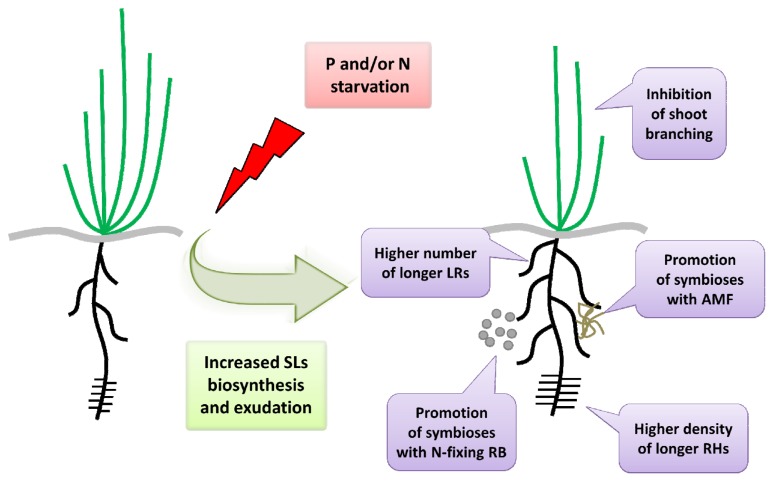
Plant responses to P and/or N starvation stress mediated by an increased production and exudation of SLs.

A new role of SLs in plant root growth and development has been proposed [11,50]. An analysis of Arabidopsis mutants that were deficient in SL biosynthesis (*max3-11*) and signaling (*max2-1*) revealed more a branched phenotype of root systems that was caused by a higher number of LRs than in the wild-type (Col-0). The treatment of plants with GR24 reduced the density of LRs in Col-0 and mutants that were deficient in SL synthesis, but did not affect the lateral root formation in the *max2-1* mutant [11]. Additionally, treatment of seedlings with a synthetic SL resulted in the increased length of RHs in the wild-type plants and the synthesis mutants of SLs, while the mutants with a disturbed signaling pathway (*max2-1*) were insensitive to the applied hormone. Nevertheless in the case of absence of GR24 no differences in RH phenotype between mutants and wild-type plats were observed [11]. An analysis of strigolactone-deficient and -insensitive Arabidopsis mutants also revealed a reduction in the length of PRs, which was caused by a lower number of meristematic cells [50]. Moreover, it was proven that SLs may suppress the formation of adventitious roots (ARs) in pea and Arabidopsis [51]. All of these results suggest that, under control conditions, SLs have a positive effect on RH elongation and PRs growth. On the other hand, these hormones negatively control the formation of LRs and ARs (Figure 2). Nevertheless it seems that the role of SLs in modulating the architecture of root depends on cross-talk with other hormones, such as auxin. Thus, strigolactone effect could be positive or negative depending on auxin concentration [50].

There are at least three recently published papers describing the potential role of SLs in the response to P starvation in the below-ground part of plants [50,52,53]. An analysis of Arabidopsis roots under a low P level showed that in Col-0 the number of RHs increased in comparison to plants that were grown in a high concentration of P, whereas in both *max2-1* (signaling) and *max4-1* (biosynthesis) mutants, the density of RHs remained unchanged after 48-hours post germination (hpg) regardless of the P concentration and was similar to that of the wild-type plants in control P conditions [52]. When seedlings were additionally treated with GR24, the density of RHs in *max4* under a low P level increased to the value observed in Col-0, while in *max2* the number of RHs remained unchanged. In the case of SL-deficiency mutants, the exogenous hormone restores the wild-type phenotype, whereas in the case of insensitive mutants, no effect was observed. On the other hand, wild-type plants treated with the AbamineSG, that may inhibit the SLs production via abscisic acid [54], showed a decreased density of RHs in wild-type plants that were grown in a low P concentration. After treatment with AbamineSG, the number of RHs that were produced was comparable to the density of RHs observed in the non-treated SLs mutants. But all these effects were observed only in a single timepoint of experiments (48 hpg), and was not observed earlier (24 hpg), or later (72 hpg), what indicates that SLs may play transient role in this process [52].

The second aspect of the adaptation of the root architecture to P starvation is an increase in the number of LRs that can be observed in different species [48]. In Arabidopsis, the density of LRs increased almost 4 times under a P deficiency, which was correlated with a decreased number of LR primordia that started to outgrow [50]. In the case of *max* mutants under control conditions, a higher number of LR primordia was observed, which indicates that endogenous SLs may prevent the outgrowth of LRs [50]. However, it should be kept in mind that the initiation of the development and elongation of LRs in plants is also regulated by other classes of hormones, such as auxin or cytokinin and that these hormones are also involved in the plant response to a P deficiency [55].

## 5. SLs Regulate the Expression of PSI Genes

The plant response to a low P concentration includes the modification of the shoot and root architecture as well as different molecular mechanisms that are involved in the acquisition and mobilization of P in plants during the adaptation of a plant to stress [56]. An analysis of plant responses to P deficiency at the molecular level allowed a group of genes induced by phosphate starvation (PSI), mainly encoding P transporters [57], to be distinguished. A comparable analysis of PSI gene expression in wild-type and *max* mutants of Arabidopsis revealed a reduction in the efficiency of a response to nutrient stress conditions in the case of SL mutants [52]. In the case of the five genes that were examined, the induction of expression under P starvation was lower in the *max2-1* mutant in comparison to Col-0. These results were obtained for *ACP5* (encoding APase), *IPS1* (transcription factor) and three P transporters (*PHT1;2*, *PHT1;4*, *PHT1;5*), all of which are highly involved in the mobilization of P in plants in nutrient stress [58]. During the response to P deficiency, a lower expression of the *ACP5* and *PHT1;4* genes was observed in the *max4-1* mutant, which indicates differences in the response to P starvation between signaling- and biosynthesis-related SL mutants; however, in both of the cases, this response is reduced in comparison to the wild-type [52]. The role of SLs in the induction of PSI gene expression was also confirmed after treatment with GR24 and AbamineSG. The exogenous SLs increased the expression of the *PHT1;4* and *PHT1;5* genes in the *max4-1* mutant, while the inhibitor of SLs biosynthesis caused a lower induction of *ACP5*, *PHT1;4*, *PHT1;5* in the wild-type under P stress [52].

## 6. SLs Secretion in Interactions with Fungi in the Nutrient Stress Response

The history of studies on SLs began over 60 years ago with a search for the factor that was exudated by plant roots and that induced the germination of the seeds of parasites [59]. Further researches allowed SLs to be described as signal molecules that were exudated by roots into the rhizosphere for communication with different organisms such as other plants, bacteria and fungi [1,2,14]. One of the most important interactions between roots and rhizosphere organisms are the mycorrhizae symbioses between plants and AMF. Fungal hyphae penetrate root tissues during mycorrhizae, thereby increasing the capacity of soil exploration through the fungal mycelium and providing an additional source of macro- and micronutrients for plants, especially P. On the other hand, plant roots provide the fungal partner with carbohydrates [60]. However, it was shown that AMF may also take up the inorganic nitrogen from the soil and transfer ammonium and nitrate to the plant partner [61]. This symbiosis is widespread in the plant world and was established at an early stage of evolution during the conquest of land by plants. It is estimated that more than 80% of land plants formed the mycorrhizae and that the mechanisms of this process was established more than 400 million years ago [62]. The role of SLs in the interactions between plant roots and AMF was proposed [2]. The 5-deoxystrigol isolated from the *Lotus japonicus* root exudate was described as a stimulating factor for the hyphal branching of the fungal partner [2]. The promotion of AMF growth and branching is caused by the stimulation of cell proliferation and the boosting of the mitochondrial metabolism of the fungal partner by SLs [63–65]. Analysis of *Petunia hybrida* mutants *pdr1* carrying lesions in the gene encoding SLs transporter, revealed the additional role of SLs in stimulation of intracellular hyphal growth within the roots [34]. A detailed analysis after GR24 treatment revealed an increased AMF colonization while mutants with a disturbed SL biosynthesis showed a reduction of symbiosis [4,5,34,66]. Additionally, plants that have already established cooperation with fungi produce less SLs in comparison to non-colonized plants, as was described in tomato [67]. All these data confirm the crucial role of SLs during initiation of AM symbioses (Figure 2).

In order to explore the role of SLs in the regulation of mycorrhizae, the pea mutants *rms1* and *rms4* were analyzed in order to test their ability to establish interactions with AMF [42]. Surprisingly, the lack of AMF colonization was observed not only in the SLs synthesis mutant (*rms1*), but also in the case of *rms4*, the SLs insensitive pea mutant. This observation cannot be explained by a lower level of SLs in the tissue and/or root exudate because the production of this hormone in *rms4* is not different than in wild-type plants. A hypothesis about the endogenous role of SLs during mycorrhizae has been proposed. It is suggested that the perception of SLs in plant tissues is involved in the promotion of symbioses with AMF [42]. Similar results were obtained for the rice SLs insensitive mutant *d3-1* carrying lesions in the gene encoding F-box protein, the orthologue of pea (*RMS4*) [42]. Another rice mutant impaired in SL signaling, *d14-1*, showed an enhanced colonization of AMF, which was correlated with a higher production of SLs [68]. These results clearly indicate that some parts of the SL signaling network represented by the F-box protein (D3/RMS4) is required for effective AMF colonization as well as for SL perception. Perhaps symbiosis with fungi may be regulated by SL, but independent of D14 component, or D3 may recognize additional, currently unknown, chemicals involved in AM colonization [68].

Experiments carried out on pea plants that grew under low, medium and high P concentrations confirmed that the exudates obtained from roots after P starvation increased the branching of *Gigaspora rosea*, the fungi partner of pea, in comparison to the exudates of control roots [69]. The number of hyphae increased almost 3-fold and similar results were observed after treatment of fungi with GR24. Additionally, the combination of treatment with GR24 and exudate from the roots of plants grown in a low P level showed an additive effect; the rate of fungi branching was almost 6-fold higher in comparison to the control conditions [69]. However, the level of P influences the symbiosis between plant roots and AMF not only through SLs; a pea SL-biosynthesis mutant also revealed a stronger induction of the fungal partner under a P deficiency [42]. This means that SLs are not required for the enhancement of AMF colonization in response to nutrient stress conditions; however, without a doubt SLs are involved in this process [42,70]. Additional evidence for the correlation between SLs and P in the regulation of mycorrhizae was provided by an analysis of petunia plants grown in a high level of P, which showed a 50% reduction of fungi colonization in comparison to non-treated plants [71]. Additionally, microarray experiments revealed that the expression of the gene involved in the biosynthesis of SLs in petunia (*DAD1*) was repressed under high P conditions. The obtained data indicate that a low P level may induce the transcription of genes encoding enzymes that produce SLs, which are responsible for the promotion of mycorrhizae. However, treatment of plants grown at a high level of P with GR24 did not increase AMF colonization, which indicates that SLs are not the only factor regulating mycorrhizae [71]. All of the presented data have shown that the symbiosis between plant roots and AMF under P starvation is a multi-level interaction that is regulated by different mechanisms, including the production, exudation and transport of SLs [34,42,65,69,71] (Figure 2).

## 7. SLs Secretion in Interactions with Bacteria in the Nutrient Stress Response

Plant roots may interact not only with the fungi in the rhizosphere, but also with other microorganisms. Legumes are a group of plants that developed the strategy of N uptake via symbioses with N-fixing rhizobial bacteria, which are housed in special root nodules [72]. This interaction is evolutionarily younger than mycorrhizae and was probably established 60 million years ago [73]. In the case of nodulation, bacteria fix N and provide it to plants, while bacteria receive carbohydrates from plants, similar to fungi during mycorrhizae. Nevertheless, legumes may interact with both, AMF and rhizobial bacteria, in order to achieve optimal growth conditions [74].

An analysis of *Medicago sativa* plants showed that GR24 increased the number of nodules, but that this promotion of nodulation was not caused by any influence on bacteria growth or metabolism [75]. A genetic experiment conducted in the pea *rms1* mutant, in which SL biosynthesis is inactive, clearly pointed to the important role of SLs in nodulation. In control conditions, the number of nodules in the *rms1-1* mutant was almost 40% lower than in wild-type plants and the application of GR24 increased the nodulation in both [14]. A grafting experiment clearly indicated that the reduction of the number of nodules in *rms1* was due to an SL-regulated process that occurs in roots, but was not due to root morphology [14]. The final question was whether the increased exudation of SLs by legume plants occurred in response to an N deficiency [39,41]. To answer this question, the SL synthesis mutant *ccd8* was analyzed in terms the stimulation or inhibition of nodulation under different N conditions. The results of the assay indicated that SLs are not required for nitrogen to regulate nodule number, although they do promote nodulation [42].

All of the presented data confirm that under P and N starvation SLs are exuded into the soil and act as signal molecules that increase the symbioses with AMF and N-fixing rhizobial bacteria in order to obtain an additional source of the macronutrients (Figure 2). Therefore, SLs play a crucial role in the plant response to a deficiency of macronutrients; however, other factors are also involved in the initiation and stimulation of root colonization by fungi and bacteria.

## 8. SLs in Responses to Other Stresses

The role of SLs in the response to salt stress was proposed. As symbioses with AMF may also increase plant resistance to soil salinity, lettuce plants grown under a high concentration of NaCl were analyzed in terms of the exudation of SLs [76]. In the case of non-mycorrhizal plants, the exudation of strigolactones was lower than in the control under salt stress. However, SL exudation increased more than 5-fold in response to salinity when AMF was present in the medium. Since salt stress negatively influences the growth and symbiotic capabilities of AMF, a higher exudation of SLs in these conditions may be necessary in order to ensure an adequate level of mycorrhizae interactions [76]. However, this work was based only on the activity of root exudate in stimulation of parasite seed germination, so additional research on other species and mutants, using different tools, is necessary to confirm the role of SLs in the response to salt stress. Then, the model of this response, including the roles of SLs and other hormones, may be proposed. Present knowledge indicates that abscisic acid (ABA) plays a primary role in the response to salt stress and SLs may be secondary players in the plant reaction to salinity.

The defense and adaptation strategies of plants to abiotic and biotic stresses are based mainly on the redox signaling network [77]. Plant hormones regulate the production of reactive oxygen species (ROS), which are the secondary messengers that modulate the cell metabolism and/or physiology and activate the defense genes [78]. One possibility is that SLs may also influence the production of ROS and thereby may participate in the response to many different stresses [77]. The delayed senescence of leaves in Arabidopsis mutant *max2*, that is more tolerant to the oxidative stress in comparison to the wild-type [79,80], makes it useful for investigating of interactions between the SLs and redox signaling networks [81]. Moreover, carotenoids, the precursors of SLs, play a crucial role in the protection of the photosynthetic apparatus against toxic ROS and in the stabilization of the membrane lipids layer [82,83]. Since carotenoids are produced and converted during the biosynthesis of SLs, it is possible that these hormones may also be involved in the plant defense mechanisms. It has also been shown that under light stress the expression of Arabidopsis *MAX3* and *MAX4* genes, which encode the proteins that are involved in the transformation of carotenoids and the production of SLs, remained unchanged [84].

## 9. Conclusions and Perspectives

SLs are a group of plant hormones that have been intensively investigated during the last years. Each year new roles of SLs are discovered. In this manuscript, the potential functions of SLs in response to nutrient stress were discussed. Present knowledge indicates that SLs comprehensively influence plant development allowing the plant to adapt to the nutrient deficiency.

Many different aspects of SLs’ functions in plants are being intensively investigated. One of the powerful environmental factors, that affects plant growth and shoot branching, is light. First reports describing correlation between light and SLs have already been published. In 2010 analysis of microarray data indicated that SLs have a positive effect on expression of light harvesting genes in tomato [85]. SLs have also been suggested to be positive regulators of other light-associated processes involved in the root and shoot development [86,87]. Furthermore, the participation of SLs in the senescence has been proposed, based on the observation of some SLs mutants showing delayed leaf senescence [47,88,89]. Nevertheless functions of SLs in this process remain unexplored at this moment.

Every year of research on SLs reveals new enzymes involved in their production and players of SL perception. Research in the next few years should shed new light on the SLs biosynthesis, signaling pathways, and the functions of these hormones in plants, as well as the cross-talk with other plant hormones.

## Figures and Tables

**Table 1:** A set of the gene encoding proteins involved in the biosynthesis and signaling of SLs.

Protein	Gene				Process
	Arabidopsis	Rice	Pea	Petunia	
Iron-containing protein	*AtD27*	*D27*			Biosynthesis
CCD7	*MAX3*	*HTD1/D17*	*RMS5*	*DAD3*	
CCD8	*MAX4*	*D10*	*RMS1*	*DAD1*	
cytochrome P450	*MAX1*				
F-box protein	*MAX2*	*D3*	*RMS4*		Signaling
α/β hydrolase	*AtD14*	*D14/D88/*	*HTD2*	*DAD2*	

**Table 2:** Production of SLs under P and N deficiency.

Species	Low level of P		Low level of N		Ref.
	
	SL name	Change	SL name	Change	

**Non-legume plants**					
*Sorghum bicolor* L. (cv. Hybrid)	5-deoxystrigol	30-fold	5-deoxystrigol	30-fold	[40]

*Calendula officinalis* L. (cv. Super)	Orobanchol	++	Orobanchol	+	[41]

	Orobanchyl acetate	++	Orobanchyl acetate	+	

*Triticum aestivum* L. (cv. Chinese Spring)	Orobanchol	++	Orobanchol	+	[41]

*Lactuca sativa* L. (cv. Chirimensha)	Orobanchol	++	Orobanchol	+	[41]

	Orobanchyl acetate	++	Orobanchyl acetate	+	

*Solanum lycopersicum* L. (cv. MoneyMaker)	Orobanchol	+	na	na	[45]

	Solanacol	+	na	na	

*Solanum lycopersicum* L. (cv. M82)	Orobanchol	100-fold	Orobanchol	-	[41]

*Oryza sativa* (cv. IAC 165)	Orobanchol	++	Orobanchol	++	[44]

	2′-epi-5-deoxystrigol	++	2′-epi-5-deoxystrigol	++	

*Oryza sativa* (cv. TN 1)	Orobanchol	+	orobanchol	+	[44]

	2′-epi-5-deoxystrigol	+	2′-epi-5-deoxystrigol	+	

**Legume plants**					

*Trifolium pretense*	Orobanchol	20-fold	Orobanchol	-	[39]

*Medicago sativa* L. (cv. BRS511)	Orobanchol	+	Orobanchol	-	[41]

	Orobanchyl acetate	+	Orobanchyl acetate	-	

*Astragalus sinicus* L. (cv. Pinkyfield)	Sorgomol	14,000-fold	Sorgomol,	1000-fold	[41]

	5-deoxystrigol	1000-fold	5-deoxystrigol	20-fold	

Pisum sativum L.	Fabacyl acetate	10-fold	Fabacyl acetate,	3-fold	[42]

	Orobanchyl acetate	10-fold	Orobanchyl acetate	3-fold	

Data described in the text; na, not analyzed; +, increased in comparison to the control plant; ++, increased in comparison to the other cultivar.
